# Is sex still binary?

**DOI:** 10.1515/medgen-2023-2039

**Published:** 2023-08-16

**Authors:** Christoph Rehmann-Sutter, Olaf Hiort, Ulrike M. Krämer, Lisa Malich, Malte Spielmann

**Affiliations:** Universität zu Lübeck Institut für Medizingeschichte und Wissenschaftsforschung Königstraße 20 23552 Lübeck Deutschland; Universität zu Lübeck Klinik für Kinder- und Jugendmedizin, Sektion für Pädiatrische Endokrinologie und Diabetologie Ratzeburger Allee 160 23538 Lübeck Deutschland; Universität zu Lübeck Klinik für Neurologie Ratzeburger Allee 160 23538 Lübeck Deutschland; Universität zu Lübeck Institut für Medizingeschichte und Wissenschaftsforschung Königstraße 20 23552 Lübeck Deutschland; University Medical Center Schleswig-Holstein, University of Lübeck & Kiel University, Institute of Human Genetics Ratzeburger Allee 160 23562 Lübeck Deutschland

## Abstract

In this perspective article we discuss the limitations of sex as a binary concept and how it is challenged by medical developments and a better understanding of gender diversity. Recent data indicate that sex is not a simple binary classification based solely on genitalia at birth or reproductive capacity but encompasses various biological characteristics such as chromosomes, hormones, and secondary sexual characteristics. The existence of individuals with differences in sex development (DSD) who do not fit typical male or female categories further demonstrates the complexity of sex. We argue that the belief that sex is strictly binary based on gametes is insufficient, as there are multiple levels of sex beyond reproductivity. We also explore the role of sex in sex determination, gene expression, brain development, and behavioural patterns and emphasize the importance of recognizing sex diversity in personalized medicine, as sex can influence disease presentation, drug response, and treatment effectiveness. Finally, we call for an inter- and transdisciplinary approach to study sex diversity and develop new categories and methodologies that go beyond a binary model.

## Introduction

The concept of sex as “binary”, meaning that there are two and only two distinct categories of male and female, is deeply ingrained in many societies. It is a concept that seems to “work” well in many respects and has been perpetuated through science, religion, and culture. However, this concept has been challenged by medical developments and scientific insights as well as a growing understanding of gender diversity in society. The legal recognition of the gender identity of non-binary individuals is an expression of acknowledgment and respect of gender diversity. In recent years, the idea that biological “sex” is entirely natural, unaffected by history and independent from language and socio-cultural categories has lost credentials. As a result, the question of whether sex *is* still binary is a topic of debate.

To understand the complexity of this question, it is important to define what is meant by “sex.” Sex refers to biological characteristics, but this includes many levels: chromosomes, gametes, hormones, genitalia, and secondary sexual characteristics such as breast development or facial hair. Historically, sex has been viewed as a binary, with individuals being classified at birth as either male or female based on their genitalia. However, the reality of embodiment is more complex than a simple binary classification would allow it to be. 

Individuals with differences of sex development (DSD) are born with variations in their sex characteristics that do not fit typical male or female categories. This may include differences in genitalia, hormones, or chromosomes. According to conservative estimates [1], around 1 in 5,000 babies are born with atypical sex traits. DSD is a family of rare but regularly occurring phenomena. The characterization of genetic and hormonal composition of DSD people’s bodies has taught us much to improve our understanding of sex development in terms of regulative networks rather than as strict binary switches. As we begin to understand the complexity of sex (and gender), it becomes clear that the binary classification of sex was a rather normative presumption. DSD embodiments need to be described empirically without *a priori* assuming oversimplified sex categories. 

In this perspective article we highlight the recent discussion about the limitations of the concept of sex as a purely binary concept and argue for an inter- and transdisciplinary approach to study sex diversity. 

## The challenge of binarity 

One still dominant view on the diversity of expression patterns is that sex is ontologically bound to reproductive functions, and therefore to sexual organs and gametes. Since there are only two kinds of functioning gametes (oocytes and spermatocytes), therefore sex must necessarily be reduced to two mutually exclusive categories – male and female. As simple as this line of reasoning may seem, it can be called into question when considered carefully. 

**Figure 1:**
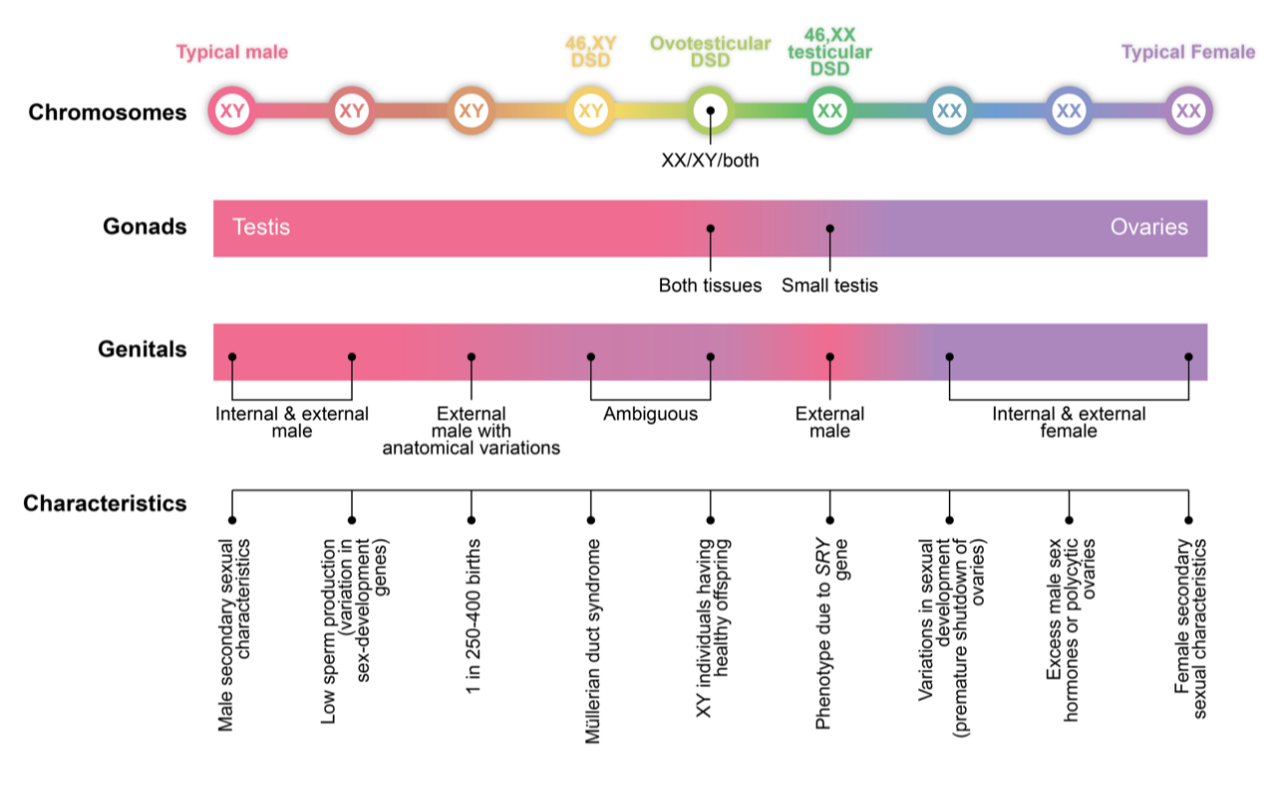
The sex spectrum (modified from Ainsworth, 2015)

There are at least two reasons for the belief that the inference from the binarity of gametes to binarity of sex is *not* justified, namely the argument (i) from somatic complexity and (ii) from evolution. 

(i) The argument from somatic complexity is the following: From the assertion that there are two kinds of gametes, it is *not necessary* to conclude that there is no variety of embodiments of sex beyond male and female. If we stick to the definition of sex given above (sex refers to biological characteristics, including chromosomes, gametes, hormones, genitalia, and secondary sexual characteristics such as breast development or facial hair), we need to take more than one phenomenal level into account than only gametes. Since infertile individuals also belong to the human species and are biologically assigned to sex categories, it is possible that human individuals can be sexed even without being able to produce fertile gametes. Gametes therefore cannot be claimed to be ontologically superior to define a sex, overriding all other phenomenal levels. On other phenomenal levels, there are indeed more than two types. There are individuals whose sex chromosomes do not match their sexual anatomy. There is a broad range of DSD embodiments. The genetic, endocrinological and anatomical levels can vary, in part independently from each other, resulting in a spectrum of possible embodiments (Figure 1) [2]. Therefore, the comprehensive phenomenon of sex is *not necessarily* binary.

(ii) Another argument is rooted in evolutionary thinking: For reproduction to be possible, it is sufficient that a *majority* of the individuals are fertile females and males (defined according their gametes and reproductive organs). Evolution therefore can accommodate the possibility of a minority of phenotypic variations other than females and males, as long as a sufficient part of individuals are fertile females and males engaged in reproduction. In addition, some individuals diagnosed with DSD are also partially fertile and can produce offspring. Hence, the argument that it would not be possible for evolution to take place if sex is not binary, is moot. 

Both arguments demonstrate that from the fact of two types of gametes there is no valid direct conclusion to the binarity of sex as a comprehensive biological phenomenon. They support the belief that (biological) sex can be seen as more complex than binarity assumes it to be. Consistent with empirical scientific evidence we can see sex as the bodily reference to “gender”, which is the term for a socially defined category system. As gender is historically disentangled from strict binary assumptions, also sex *can* be seen as having more possible variations, albeit rare, than only two. 

This allows to better integrate bio-scientific knowledge about “sex” and socio-cultural knowledge about “gender” in all important aspects of sex-determination. And it opens new research perspectives, some of them transdisciplinary. The processes of sex diversification do not necessarily need to be parallel on all levels of development, and how these different levels interact will be important to study. They range from karyotype, epigenetics, the metabolome to psychological development, emergence of sexual desire and gender identification, to social constructions of gender and societal gender governance.

## What do we know so far?

### Sex development and expression 

Sex determination is regulated by a molecular antagonism between testes and ovary developmental pathways. The interaction of the genetic pathways for gonadal determination is currently seen as a balance in a regulatory network between different genes that promote testicular or ovarian development, thereby already challenging a “black and white” perception between “male” and “female” development. It has recently been shown that regulatory rearrangements can lead to an adaptive intersexuality in moles. In chromosomally female moles, a tandem triplication involving the *CYP17A1* gene and an intrachromosomal inversion affecting the *FGF9* gene cause the development of ovotestes and subsequent androgenisation, which may lead to better survival strategies based on increased muscle mass and further effects of endocrine action to enhance strength and attentiveness in rodents [3]. 

Androgenic steroids lead to irreversible downstream expression patterns of genes corresponding to induction of the male phenotype in cells of the genital tubercle. Thus, these androgenic steroids shape the cellular transcriptome and epigenome in the developing embryo in a “male programming window” [4]. We must presume that androgenisation, and possibly oestrogenisation, also exerts its effects on body composition in factors such as muscle/fat ratio, bone mineral density etc. Further (prenatal) effects of oestrogenic steroids remain to be studied. Data from a recently published 46,XX patient with gonadal dysgenesis suggest that a functioning oestrogen receptor beta is needed for proper ovarian development [5]. However, interestingly, the most androgenic steroid, dihydrotestosterone (DHT), is mainly not synthesized in the testis, but rather a peripherally converted metabolite of testosterone. Moreover, DHT can be synthesized through alternative pathways and sufficient amounts of DHT will lead to an androgenisation and virilisation of any individual regardless of the gonadal or even chromosomal composition. The lesson was learned from people with DSD conditions, where an individual with deleterious mutations in SRD5A2 will lack DHT synthesis and develop an initial female appearing phenotype despite functioning testes and a 46,XY karyotype. In contrast, 46,XX individuals with 21 hydroxylase deficiency leading to congenital adrenal hyperplasia will virilise due to DHT excess via a backdoor pathway synthesis [6].

Little is known about the role of oestrogens in prenatal sex development. From patients with complete gonadal dysgenesis, who presumably have no gonadal function and should therefore lack systemic oestrogen synthesis, we know that sex development is comparable to other 46,XX females. However, the foetus is usually exposed to oestrogenic steroids via the placenta and should therefore receive some general effects through these compounds. Some endocrine disrupting compounds, such as bisphenol A, are actually actively enriched in the foetus. There are recent reports of the effects on fertility and sexual development of people previously exposed as foetuses [7]. At the level of gonadal development, the oestrogen receptor beta may play a role. In this context, mutations of the *ESR2* gene have been documented in both 46,XY DSD and 46,XX ovarian dysgenesis [8,5]. The pathways and effects of the endocrine system in sex development are not yet fully understood. The presumption that only specific hormones such as androgens or oestrogens determine the sex phenotype should be replaced by a concept of profiling sex hormones that lead to the variability of phenotypic expression in a time-, compound- and dose-dependent manner.

### Individual sex phenotype

Body shape and proportions are modulated through an intricate interplay of genetic and endocrine/metabolic factors that correspond to an individual sex phenotype. All in all, sex-dependent gene expression occurs in almost all tissues and affects a broad range of body functions, far beyond sexual and reproductive functioning [9]. 

This also includes the brain, which is very interesting in terms of variations in sex development, because it influences gender identity, gender role behaviour and sexual orientation. Psychological aspects of sex develop through biological factors (genes and hormones) as well as through psychological, social and cultural factors. To some extent, sex-specific gene expression is already present in the brain before gonadal differentiation sets in [10]. The influence of perinatal androgens on the sex-specific development of certain brain regions in rodents has been known for 40 years [11]. In humans, a lot of research on sex differences in human brain structure and function followed a binary concept of sex. However, the evidence speaks against a simple sex dimorphism in macroanatomical brain structure [12].

Sex steroids play an activating and organisational role in the brain and in behavioural development. While activating influences are transient and correspond to a waxing and waning of hormone levels, e. g. associated with sexual interest, organisational aspects are permanent and will, as in sex determination and development in general, persist throughout life [13,14]. At the same time, however, there is a great deal of scientific debate about how far these endocrine consequences really go and the extent to which gender stereotypes feed into behavioral research where they lead to a bias of “neurosexism” [15]. 

### Behavioural patterns

Perinatal hormone exposure may influence behavioural patterns. In male rats, both androgen and oestrogen treatment in the perinatal period induce male-specific behaviour in adulthood. Interestingly, in the rat brain testosterone is converted into oestrogen through aromatase, and this is thought to masculinise specific brain regions irreversibly, including the sexually dimorphic nucleus of the preoptic area [16]. More recent studies have shown that androgen action via the AR in the early postnatal period is also critical for brain masculinisation in the rat [17]. The masculinising effect of oestrogens on the rat brain has also been seen in female rats treated neonatally with 17β-oestradiol, leading to a masculinised perioptic area and sexual behaviour in adulthood [18]. In the female rat, steroid hormones have been shown to interfere with DNA methyltransferases (Dnmts), which are needed to repress masculinising genes in the preoptic area. Treatment with Dnmts inhibitors led to masculinised sexual behaviour in female mice, indicating active suppression of masculinisation via DNA methylation in the female rat [19]. The prenatal influence of steroid hormones in humans is still largely unknown, but both oestrogens and androgens have been shown to influence human behaviour postnatally [14, 20]. Importantly, this research shows that the development of brain and behaviour does not depend only or mainly on the genetic composition of an organism, but rather on the hormonal makeup in all its complexity and variability. Beyond hormones, of course, a great many other biological, social and psychological factors, including gender roles, play a role in shaping individual behavior

### The role of sex diversity in personalized medicine 

“Gender medicine” deals with differences between the “male” and “female” organism. It has been perceived as a first step towards personalised medicine and patient-centred care. Indeed, one of the primary ways in which sex impacts modern medicine is through its effects on disease presentation. For example, studies have shown that men and women often exhibit different symptoms when presenting with the same disease, and that these differences can have a significant impact on diagnosis and treatment [21]. In addition, certain diseases are more prevalent in one sex than the other, and understanding these sex-specific differences is crucial for developing effective prevention and treatment strategies. Sex differences also play a significant role in drug metabolism and response [22]. Furthermore, research has demonstrated that the inclusion of sex-specific data in clinical trials can lead to more accurate and effective treatments. Historically, many clinical trials have only included men or have not analyzed sex-specific data. However, there is a growing recognition of the importance of including both men and women in clinical trials to ensure that treatments are effective and safe for everyone [23]. This approach is known as “sex and gender-based analysis” (SGBA), and it aims to identify and account for sex-specific differences in all aspects of health research. In recent years, there has also been a push towards personalized medicine, which considers individual differences, including sex [24]. By recognizing sex as a variable in diagnosis and treatment plans, healthcare providers can provide more effective and equitable care to their patients. However, gender medicine mostly assumes a purely binary gender model, not considering the diversity of sex and gender. Truly personalized medicine needs to account for the diversity of sex in its different biological levels (genetic, hormonal, etc.) and its impact on medical diseases and treatment. In line with the ambition of personalised medicine and patient-centred care, new categories – and accordingly adapted methodologies of health research– need to be developed for patients who cannot be accommodated by a binary scheme [25]. Studying the effects of sex differences in health and disease will lead to new treatments that target sex hormone and sex-chromosome effects. These will ultimately help people irrespective of their sex [23, 26].

### The role of DSD individuals in medicine 

Differences of Sex Development (DSD) describe a heterogeneous group of humans with discrepancies between chromosomal, gonadal and phenotypic sex [27]. It has long been recognized that people with DSD conditions are part of our societies, requiring special attention for several aspects [28]. The clinical findings of conditions that affect sex development and maturation can be highly variable and sometimes clinically undetectable. This clinical observation holds true for conditions such as complete gonadal dysgenesis (where the external phenotype is female, even if the karyotype is 46,XY) and in patients who are 46,XX and have congenital adreanal hyperplasia (CAH; who may be virilised at birth but in due course often follow the female patterns). Thus, we have learned many aspects on the heterogeneity, timing and variability of sex development from investigations of people with DSD conditions. Very recent work of our group has demonstrated that DSD is a model system that allows novel and exciting insight into development of sex diversity in gonads [29], in the protein-protein interaction of androgen signalling [30], and also in the overarching aspects of care for people with rare and complex conditions in a changing society [31].

Historically, medical interventions for DSD have been aimed at “normalizing” genitalia and promoting binary gender identity. However, these interventions have been controversial, as they may not be necessary for the health and well-being of the individual and can lead to negative outcomes, such as loss of sexual sensation or fertility. In recent years, there has been a shift towards a more patient-centered approach to the management of DSD. This approach involves engaging patients and their families in shared decision-making and providing comprehensive, multidisciplinary care that addresses the physical, psychological, and social aspects of DSD. This approach recognizes the diversity of experiences and needs among individuals with DSD and aims to promote their autonomy and well-being. Furthermore, research has shown that individuals with DSD may have unique health concerns that require specialized care. For example, individuals with DSD may be at increased risk for certain medical conditions, such as gonadal tumors or infertility, and may require regular monitoring and screening. Additionally, individuals with DSD may experience mental health concerns, such as anxiety or depression, related to their diagnosis and experiences of stigma and discrimination. In conclusion, the role of DSD in medicine is complex and multifaceted. The challenges associated with the diagnosis, treatment, and management of DSD require a patient-centered approach that recognizes the diversity of experiences and needs among individuals with atypical reproductive or sexual anatomy. By providing comprehensive, multidisciplinary care and engaging patients in shared decision-making, healthcare providers can support the health and well-being of individuals with DSD. Additionally, continued research and education on DSD are essential for advancing healthcare and promoting greater understanding and acceptance of individuals with atypical reproductive or sexual anatomy.

### Deconstruction of the sex binary in the humanities

Historians such as Claudia Honegger, Thomas Laqueur or Emily Martin in the 1990s have extensively investigated and historically deconstructed the origins of the idea of sex binarism [32, 33]. The idea that sex is organized as a binary as we know it today seem to have emerged in the 18^th^ and 19^th^ centuries anatomy and physiology. Its emergence can be explained in relation to the historical conditions of that time. Sarah Richardson’s investigations into the history of sex genetics in the 20^th^ century found that genetics at first struggled with the idea of binarism. The answer that XX and XY karyotypes explain sex in a binary way was not evident from the beginning [34]. A plausible conclusion is that the binarity of sexes is not a biological “given” but needs rather to be regarded as a result of particular socio-historical processes. 

These developments have seriously weakened the separation between sex and gender. Gender studies research demonstrated that biology is not mirroring nature with its theories but necessarily uses descriptive and interpretative concepts as well as language and linguistic categories that have their origins in culture and society. As Andrea Maihofer wrote, to connect an organ, a molecule, a chromosome or a hormone with the meaning as a sexual sign is a societal act. “Sex” chromosomes themselves have no sex. Judith Butler’s deconstruction of gender as a performance and sex as a process of materialization also relates to the binarity of the order of the sexes [35]. In sum, feminist research in the humanities have first debunked essentialism of gender norms and then also the essentialism of a binary opposition of two sexes.

## A research perspective

Inter- and transdisciplinary research on the determinants, meanings and implications of sex diversity needs a new initiative to bridge, integrate, and stimulate work in the natural sciences, in medicine, in the social sciences, law and the humanities. Research in this context needs to acknowledge that a binary model of only two mutually exclusive sexes is in conflict with both old and recent findings in biology, medicine and the humanities. This binary model is based on presumptions that cannot accommodate the dynamics and complexity of the phenomena of sex development and expression, or the diversity of experiences of sex, gender and their meanings. The binary model is just one of many possibilities to study, model and explain the relation of the sexes and to organise differences on the sex/gender axis.

**Figure 2:**
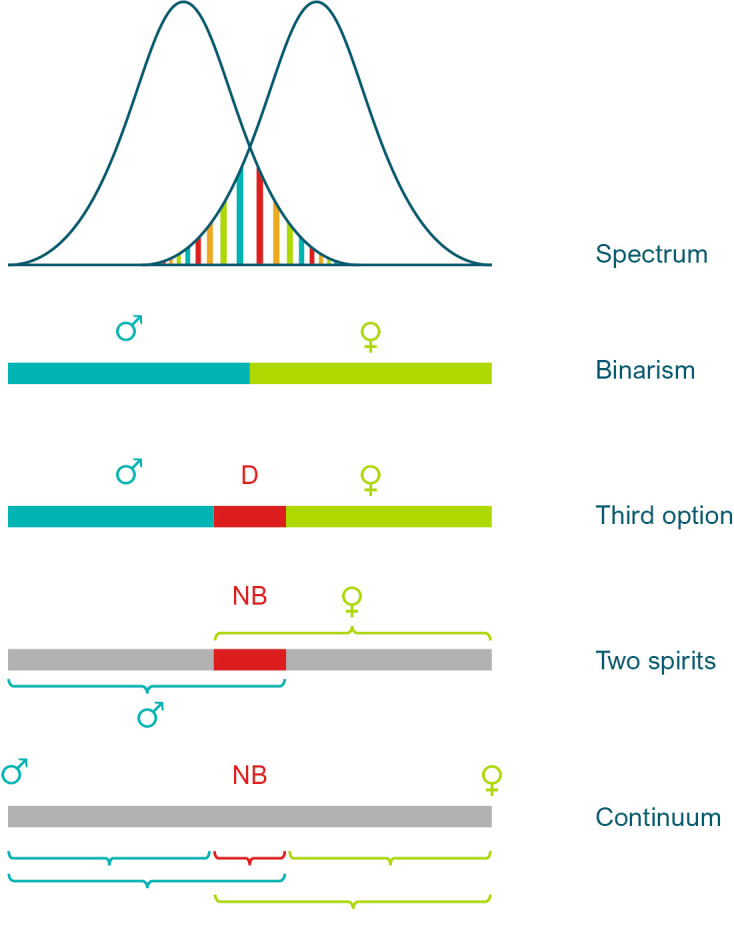
Multiple logics of seeing sex/gender diversity and (non-)binarity: a) as a spectrum with overlaps, which may constitute DSD, b) reduction to a binary on a deeper ontological level, c) with a “third option” in between, in German law called “diverse”, d) non-binary people as both sexes, or “two spirits”, e) as a continuum with multiple options to distinguish between sex/gender categories, depending on contexts.

Recognising and investigating the diversity of sexes beyond unquestioned binarity may have profound effects on understandings of sex related phenomena and also on scientific research practices as well as on wider society. The emerging understandings need to be systematically studied from diverse disciplinary angles and stakeholder perspectives, and through a multitude of interlinked methodological approaches. 

Several questions need to be addressed: What establishes the intelligibility of sex categories (beyond the two sexes) for different actor perspectives (people concerned, families, activist groups, and also from different research perspectives)? How is sex to be located as a category within the context of specific biomedical research programs? When and in what regard is “sex” a relevant category and how many categories of sex need to be specified in certain research contexts? The complexity of developmental processes and the differentiation of sex-related features has to be investigated without presuming unquestioned strict binarity. To tackle genesis and meanings of sex and gender diversity comprehensively and on all relevant levels requires consideration of interactions of socio-cultural parameters with clinical practice and experimental research.

Binarity, if it still appears in research settings, can be seen, as some scholars have suggested, as “a quick-and-dirty way to capture unmeasured, still-to-be-explained variance” [36]. Leaving behind binarity however opens up a variety of alternative ‘logics’ of distinction in the sex/gender spectrum. **Figure. 2** presents a number of possibilities, however with the limitations of two-dimensional static models. In view of the multiple dimensions of sex that coexist in one person and possible changes over time, the future lies in higher-dimensional concepts, even if this poses great challenges to science. One possibility is to consider a ‘third category’ in-between the two, which can be conceived as more or less homogenous in type, or as internally specified. Another possibility is to conceive the in-between space as an overlap of two sexes, as some traditional cultures have assumed where a social category of ‘two-sprits’ is existing [37]. From a purely logical point of view, there may also be a space of asexuality, neutrality or ‘no-sex’. This resonates with the proposal made by some political philosophers to recognize people in law solely as persons independent from their sexed existence [38, 2]. Or, the sex spectrum can be conceived as a continuum with innumerable possible distinctions apart from the two majority sexes, with contingent and context-relative categorizations. Furthermore, all sex categories used in science and culture can be conceptualized as discrete or continuous [39]. Lastly, as cultural tradition and the vast feminist discussion about sex and gender shows, the very distinction of *two* sexes can even be conceived in many ways. Patriarchal societies uphold the view that sexes are polar, mutually exclusive opposites, which are the basis of a hierarchical social order [40]. However, sexes can also be seen as variations within an open range of possible ways of existence. This will not deny the existence of the large groups of women and men. But it will perhaps challenge the way they perceive themselves as exclusive natural “givens”.
